# Unravelling the chemodiversity of eggplants - Insight into their role in the underlying response to biotic and abiotic stresses

**DOI:** 10.3389/fpls.2025.1696668

**Published:** 2026-01-05

**Authors:** Meenakshi Subramanian, Nikhil Kumar Ramesha, KP Abhiram, Manoj Kumar, Pattantavida Vismaya, Srinivasamurthy Vanishree, HS Likitha Aishwarya, Srivatsa Udupa, Swathi Shivappa, Puthanvila Surendrababu Swathy, Sachin Ashok Thorat, Arya Kaniyassery, Laura Toppino, Yu-Chung Chiang, Annamalai Muthusamy

**Affiliations:** 1 Department of Plant Sciences, Manipal School of Life Sciences, Manipal Academy of Higher Education (MAHE), Manipal, Karnataka, India; 2 John Innes Centre, Norwich Research Park, Norwich, United Kingdom; 3 Council for Agricultural Research and Economics Research Centre for Genomics and Bioinformatics, Montanaso Lombardo, Italy; 4 Department of Biological Sciences, National Sun Yat-sen University, Kaohsiung, Taiwan; 5 Department of Biomedical Science and Environment Biology, Kaohsiung Medical University, Kaohsiung, Taiwan

**Keywords:** *Solanum melongena*, chemodiversity, abiotic stress, biotic stress, secondary metabolites

## Abstract

*Solanum melongena* L. is a significant annual vegetable crop belonging to the Solanaceae family. It is cultivated worldwide, particularly in tropical and subtropical regions. It is rich in proteins and dietary fibres and contributes to its broad range of secondary metabolites, thereby increasing its chemodiversity. Secondary metabolites like phenolics, terpenoids, glycoalkaloids, flavonoids, and antioxidants act as stress regulators. While eggplant is known for its phytochemical profile associated with nutraceutical properties, the role of its chemodiversity in conferring tolerance to stresses remains underexplored. Therefore, understanding the chemodiversity of eggplant is crucial for developing stress-resistant cultivars. This approach addresses a critical gap by linking chemodiversity with adaptive responses and offers new perspectives for crop improvement. Currently, researchers are widely using metabolomics, high-throughput analytical tools and bioinformatic tools to evaluate chemodiversity in different parts of plants. Large-scale characterization of the phytochemical diversity of eggplant genotypes under various stress conditions has been performed via high-throughput screening techniques. Understanding the regulatory network and biochemical pathways involved in stress adaptation in eggplant can be accomplished by integrating metabolomics, genomics, and transcriptomics. Overall, this review discusses the importance of chemodiversity in eggplant during stress conditions by highlighting the chemical and metabolic diversity of different eggplant cultivars and their wild relatives, emphasizing their functional roles in plant defense and stress adaptation.

## 1 Introduction

*Solanum melongena*, commonly known as eggplant, brinjal, or aubergine, is a significant vegetable crop which belongs to the nightshade family, Solanaceae. With a global production of more than 58.6 million tons in 2021, eggplant is a crop of both economic and nutritional importance (Subramaniam and Kumar, 2023). Among the various crops within the Solanaceae family, eggplant is the second most commercially significant crop, followed by tomatoes (Rakha et al., 2021). Eggplant is considered a rich source of vitamins, minerals, proteins and carbohydrates and is used in various dishes worldwide. Countries such as China, India, Egypt, Turkey, Indonesia, Bangladesh, Iran, Italy, Japan, and Spain are major nations cultivating eggplant. In India, the annual eggplant production is 12 million tons on 681,000 ha (FAOSTAT, 2023, https://www.fao.org/faostat/en/#data/QCL). Eggplants are known for their various culinary and medicinal value, particularly their cardioprotective, antidiabetic, anti-inflammatory and antioxidant properties. Presence of bioactive compounds like phenolic acids, flavonoids and glycoalkaloids contribute to these medicinal properties (Contreras-Angulo et al., 2022; Sharma et al., 2024). It has also been demonstrated that some phytochemicals present in eggplant are able to induce apoptosis in several human cancer cells (Sharma et al., 2024).

As sessile organisms, plants are incapable of migrating to environments that offer more favourable conditions. Instead, they employ various responses to survive harsh conditions, one of which is the synthesis of various secondary metabolites (Jin et al., 2024). These metabolites influence certain characteristics in plants, enabling them to adapt to, regulate, and interact with their environment (Rabeh et al., 2025). To date, up to 200,000 plant secondary metabolites have been identified and characterized. These include polyacetylenes, cyanogenic glycosides, quinones, phenolic acids, terpenes, amines, glucosinolates, alkaloids, and peptides. A total of 25,000 terpenes, more than 10,000 alkaloids, 10,000 phenolic compounds, and 120 glucosinolates have been reported (Divekar et al., 2022). Chemodiversity, therefore, refers to the diverse phytochemicals produced by plants and is considered a key component of the plant phenotype. It also influences the interaction of plants with pollinators and herbivores and protects against biotic and abiotic stresses (Petrén et al., 2024; Thon et al., 2024).

Recent research indicates that our understanding of stress-responsive metabolite profiles is increasing rapidly, emphasizing their potential importance in the metabolic response of plants to stress. Stress conditions affect more than 50% of the estimated 200,000 secondary metabolites found in plants. Many of these compounds, including terpenoids, flavonoids, phenolics and alkaloids play key roles in plant defense by functioning as antioxidants, antiherbivore and antimicrobial agents (Pant et al., 2021; Muthusamy and Lee, 2024). Eggplants have been reported to exhibit extensive chemodiversity, producing a wide range of chemical compounds. This chemical diversity helps plants to adapt to various environments and defend themselves against pests and diseases.

Although multiple reports on plant secondary metabolites and their role in stress responses are available, comprehensive studies focusing specifically on the chemodiversity of eggplant and its role in mediating stress resistance are limited. In addition, only a few studies have integrated high-throughput analytical techniques to identify metabolic pathways and the genetic factors regulating them. The objective of this review is therefore to conduct an in-depth analysis of the phytochemical and metabolic diversity of eggplants, focusing on how these compounds may contribute to plant defence mechanisms against various biotic and abiotic stresses. This review also highlights recent advances in metabolomic approaches and their potential to identify key biomarkers for stress tolerance. This information will aid in the development of novel strategies for breeding more resilient cultivars and sustainable agricultural practices.

## 2 Phytochemical constituents of eggplant

The key phytochemical compounds present in eggplant are phenolic acids (chlorogenic acid), flavonoids (quercetin, rutin, lutein, naringenin, kaempferol, apigenin, isorhamnetin and luteolin) and glycoalkaloids (solasodine, α-solamargine and α-solasonine). The predominant class of phytochemicals found in eggplant is phenolic acids, with chlorogenic acid (5-O-caffeoylquinic acid) being the most predominant compound (Contreras-Angulo et al., 2022). There have also been reports on the presence of hydroxycinnamic acid derivatives such as caffeic acid, coumaric acid and ferulic acid, which confer anti-inflammatory and antioxidant properties to the fruit (Kim et al., 2025).

Nasunin (delphinidin-3−(p−coumaroylrutinoside) -5−glucoside) is the main anthocyanin found in the peel of eggplant, conferring it a violet–purple color (Florio et al., 2021; Alighadri et al., 2023). α-Solasonine and α-solamargine are the two glycoalkaloids identified in eggplant fruit that share the same aglycone (solasodine) but differ in their trisaccharide compositions. The trisaccharide of α-solamargine is chacotriose (two rhamnoses and one glucose), whereas that of α-solasonine is solatriose (one rhamnose, one glucose and one galactose) (Mauro et al., 2020). These compounds are primarily concentrated in the peel and play vital role in defense mechanisms of plants (Lucier et al., 2024).

## 3 Chemodiversity in eggplant

**Figure 1:**
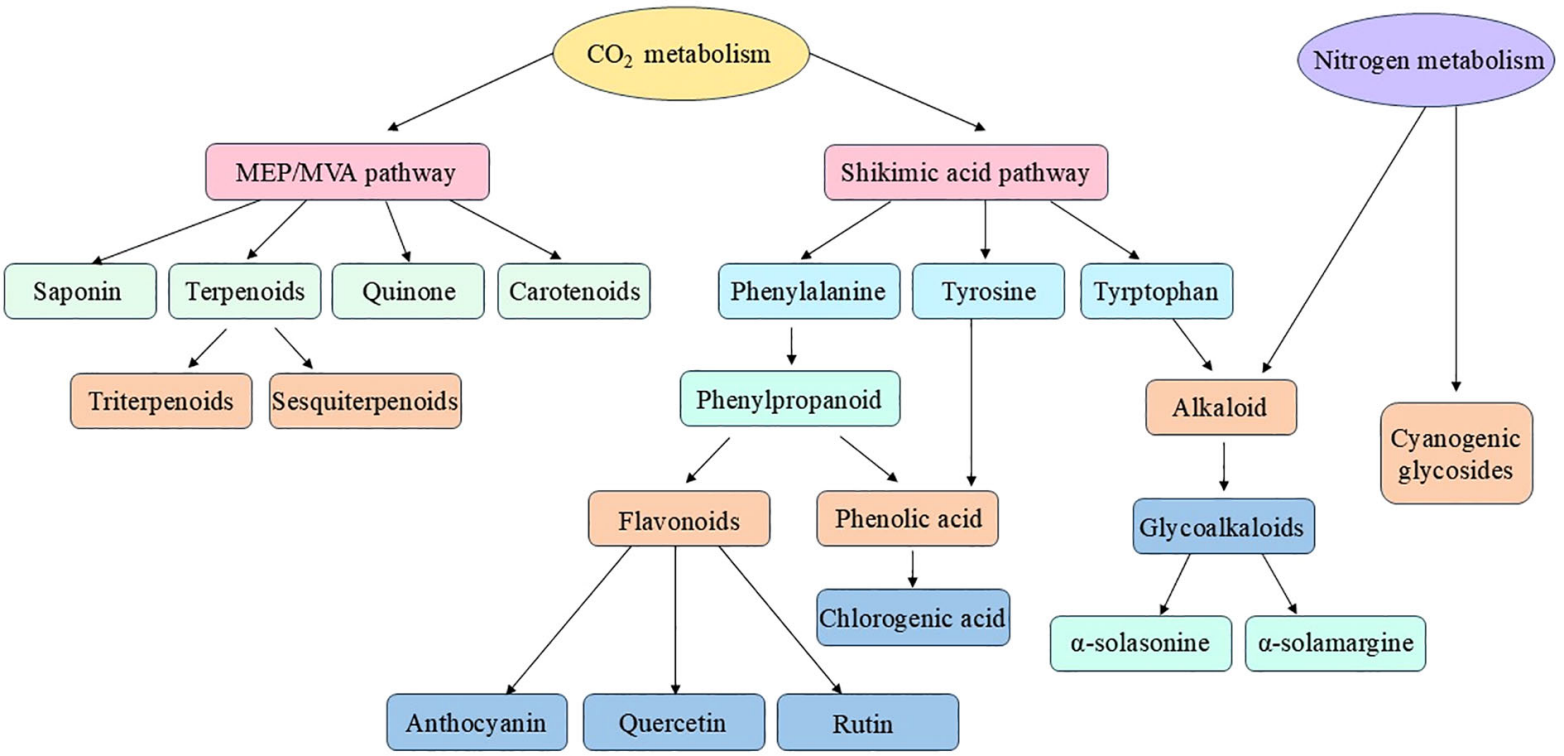
Schematic representation of the primary biosynthetic origins of key phytochemicals such as terpenoids, alkaloids, flavonoids, phenolics, saponins, and cyanogenic glycosides derived from core metabolic pathways such as the shikimate, mevalonate (MVA), methylerythritol phosphate (MEP), and phenylpropanoid pathways. The arrows represent biosynthetic relationships (modified from Böttger et al., 2018; Jamloki et al., 2021). Created via Microsoft PowerPoint.

Eggplants exhibit significant chemical diversity due to the biosynthesis of metabolites such as anthocyanins, steroidal glycoalkaloids (SGAs), flavonoids, and phenolic acids. These metabolites significantly impact characteristics such as fruit color, flavour, nutritional value and defense mechanisms (Zhou et al., 2023; Fiesel et al., 2024). Their biosynthesis involves multiple interconnected metabolic pathways, such as the methylerythritol phosphate (MEP)/mevalonate pathway (MVA), the shikimic acid pathway and the phenylpropanoid biosynthesis pathway. These pathways help in protecting plants against stress and pathogens (**Figure 1**).

Numerous secondary metabolites at varying concentrations have been observed in various parts of eggplant. Phenylpropanoid amides in eggplant can be classified into four types on the basis of their precursors, the way in which they accumulate in different organs, and variations in concentration. For example, Type I and II phenylpropanoids were detected only in eggplant leaves, stems, and roots. Fruits, on the other hand, are rich in spermidine and putrescine derivatives (type III and IV phenylpropanoids), as well as chlorogenic acid and its isomers (cryptochlorogenic acid and neochlorogenic acid). Furthermore, variations in the accumulation patterns of chlorogenic acid and anthocyanins were observed and this was attributed to the domestication of eggplant. Furthermore, compounds such as feruloylputrescine, caffeoylputrescine, coumaroylputrescine, caffeoyltyramine, and feruloyldopamine were recorded in eggplant for the first time. Though these putrescine derivatives were also found in the flesh of fruits, they were more prevalent in the peel (Sun et al., 2015).

Docimo et al. (2016) reported higher concentrations of rutin, chlorogenic acid and delphinidin-3-rutinoside in eggplant fruits than in other parts. Mauro et al. (2020) analysed the variation in phytochemicals in three genotypes of eggplant, namely, Birgah, Black Bell, and Black Moon, highlighting relevant variation in the accumulation of key compounds such as delphinidin-3-rutinoside, nasunin, and anthocyanin among these three genotypes. The Black Bell had the highest anthocyanin content in the fruit flesh and peel, followed by Birgah. The Black Moon genotype contained lowest amount of anthocyanin among the three genotypes analysed.

Zhou et al. (2023) analysed 13 eggplant cultivars (Suqie 6, Suqie 301, Suqie 9, Suqie 13, Bulita, Tewangda, Hangqie, Qinfeng, Heidabang and Liyancainuo) grown in China. These included a total of 503 primary metabolites: 69 saccharides, 156 lipids (e.g., myristic acid, hydroxylinolenic acid), 22 vitamins (e.g., vitamin B3), 92 organic acids (e.g., succinic acid, quinic acid, and malic acid), 100 amino acids (e.g., L-valine, L-aspartic acid, L-glutamic acid and L-asparagine) and 64 nucleotides, which all accumulated differently in each cultivar.

Niño-Medina et al. (2017) reported the presence of two delphinidin derivatives in the Chinese eggplant cultivar ‘Zi Chang’: delphinidin-3-glucoside-5-(coumaryl) dirhamnoside and delphinidin-3-glucoside-5-dirhamnoside. These compounds have not been detected in other eggplant cultivars. In a study carried out by Plazas et al. (2013), the accumulation of secondary metabolites in 18 different eggplant accessions was estimated. Accession V17 had the highest polyphenol oxidase (PPO) activity, chlorogenic acid content and total phenolic content whereas accession V9 had the second highest chlorogenic acid content. Although the accessions originated from a geographically limited area, the traits studied showed considerable variation, confirming the high genetic diversity of eggplant.

A comparative study on the metabolite content in the fruit flesh and peel of two varieties of eggplant (*S. melongena* var. *serpentinum* and *S. melongena* var. *esculentum*) was carried out by Niu et al. (2022). Elements such as cadmium, barium, boron, copper, rubidium, calcium, palladium, and strontium were more abundant in the peel of *S. melongena* var *serpentinum* than in the peel of *S. melongena* var *esculentum*. Furthermore, a total of 10 metabolites were found to be more abundant in the peels of *S. melongena* var *esculentum*, whereas the peel of *S. melongena* var *serpentinum* contained 52 more compounds than the peels of *S. melongena* var *esculentum*. A comparison of the metabolites present in the fruits and peels of *S. melongena* var. *serpentinum* revealed that 8 metabolites were more abundant in the fruit flesh than in the peel, whereas 39 compounds were more abundant in the peel. The peel of *S. melongena* var *serpentinum* presented increased accumulation of phenylacetic acid, L-rhamnose, quinic acid, coumaric acid, 2-methylbutyroylcarnitine, maltitol, kaempferol 3-O-rutinoside, tyramine, chlorpromazine, tangeritin and nobiletin. A total of 41 metabolites were found to significantly differ between the peel and fruit of *S. melongena* var *esculentum*. The peel of *S. melongena* var *esculentum* presented increased accumulation of 3,4-dihydroxymandelic acid, kaempferol 3-O-rutinoside, deoxycytidine, cyanidin 3-glucoside cation, tyramine, 5-methylcytosine, thymine, isoscopoletin, quinic acid and thymidine.

Comparative analysis of the phytochemical composition and antioxidant properties of three Turkish eggplant cultivars, Trabzon Kadife (white), Kadife Kemer (purple), and Aydin Siyahi (black) was carried out by Colak et al. (2022). The black cultivar presented maximum levels of total phenolics, flavonoids and antioxidant activity, followed by the purple cultivar. The white cultivar consistently presented the lowest phenolic and flavonoid contents. The predominant anthocyanin in these cultivars was delphinidin-3-O-rutinoside, and the concentration of this anthocyanin strongly correlated with the pigmentation of the peel. The delphinidin-3-O-rutinoside content was the highest in the black cultivar and the lowest in the white cultivar.

## 4 Chemodiversity and plant defense mechanisms

### 4.1 Chemodiversity and classification

**Figure 2:**
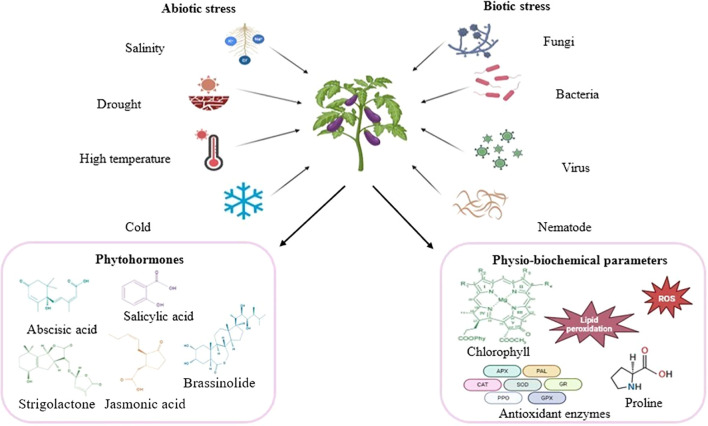
Schematic representation of the phytohormones produced and the associated physiological and biochemical responses of eggplants under abiotic and biotic stress conditions. When plants are subjected to stress, they activate signaling pathways that trigger the production of stress-related phytohormones, which help in regulating their defense mechanism. An increase in the ROS level results in oxidative damage, causing lipid peroxidation, which in turn damages the cell membrane. To reduce this damage, plants increase their antioxidant defenses and accumulate osmoprotectants such as proline. Moreover, stress affects chlorophyll production, hindering photosynthesis and overall plant health. (Modified from Shiade et al., 2024). Created via Biorender.com.

Environmental factors considerably affect the biochemical features of plants. Following exposure to abiotic and biotic stressors, the defense-related phytohormones, physiobiochemical traits and secondary metabolite contents of plants are rapidly altered (**Figure 2**). Different plant lineages produce distinct metabolites as an adaptive response to environmental changes (Wittmann and Bräutigam, 2025). This leads to diversity in the quality and quantity of these compounds both interspecifically and intraspecifically (Thon et al., 2024; Thorat et al., 2024). Chemodiversity, thus, refers to the diversity of secondary metabolites between and within plant species and can be used as a measure to estimate the chemical diversity of a population. While environmental conditions influence chemodiversity, genetic factors also contribute significantly (De Brito MaChado et al., 2024). The abundance and specific composition of secondary metabolites contribute to a plant’s chemodiversity. This variation influences plant traits, enabling adaptation, regulation, and interaction with their environment (Müller and Junker, 2022; Petrén et al., 2024). By enhancing their interaction with the environment, chemodiversity influences processes such as pollination and defense against abiotic and biotic stress (De Brito MaChado et al., 2024).

To better understand chemodiversity, it can be classified into three levels: α-, β-, and γ-chemodiversity. These levels are defined by the level at which variation in secondary metabolites is observed. α-Chemodiversity refers to the variation in secondary metabolites within the same plant; β-chemodiversity refers to the variation within a species belonging to the same population; and γ-chemodiversity refers to the variation among different plant species belonging to the same community (Ishihara, 2021). This classification provides insight into the complexity of plant metabolomes and their roles in evolution and ecology.

While the extent of metabolic variation can be determined by classifying chemical diversity, the molecular mechanisms underlying this diversity, particularly in response to stress, are equally important. Environmental stressors such as pathogens, drought, or herbivore attack trigger complex signaling networks that regulate metabolite biosynthesis.

### 4.2 Molecular mechanisms underlying the stress-induced response

Plants possess specialized receptors that facilitate their detection of external stimuli and subsequent responses to their environment. Receptor-like kinases (RLKs), which act as pattern recognition receptors (PRR), are essential for identifying environmental stress signals. When sensing external stress signals, receptor-like kinase (RLK) triggers receptor-like cytoplasmic kinase (RLCK), which activates pattern-triggered immunity (PTI). This includes activation of guanine nucleotide-binding proteins, ubiquitin, increased influx of calcium and the activation of the mitogen-activated protein kinase (MAPK) pathway, as well as the production of reactive oxygen species (ROS). The MAPK signaling pathway plays a crucial role in the transmission of these signals to the nucleus, thereby activating transcription factors such as MYBs and WRKYs and consequently resulting in the upregulation of genes involved in stress response. The observed responses include the hypersensitive response (HR), cell wall lignification, stomatal closure and the activation of ROS-scavenging enzymes such as glutathione peroxidase (GPX), catalase (CAT), superoxide dismutase (SOD).

**Figure 3:**
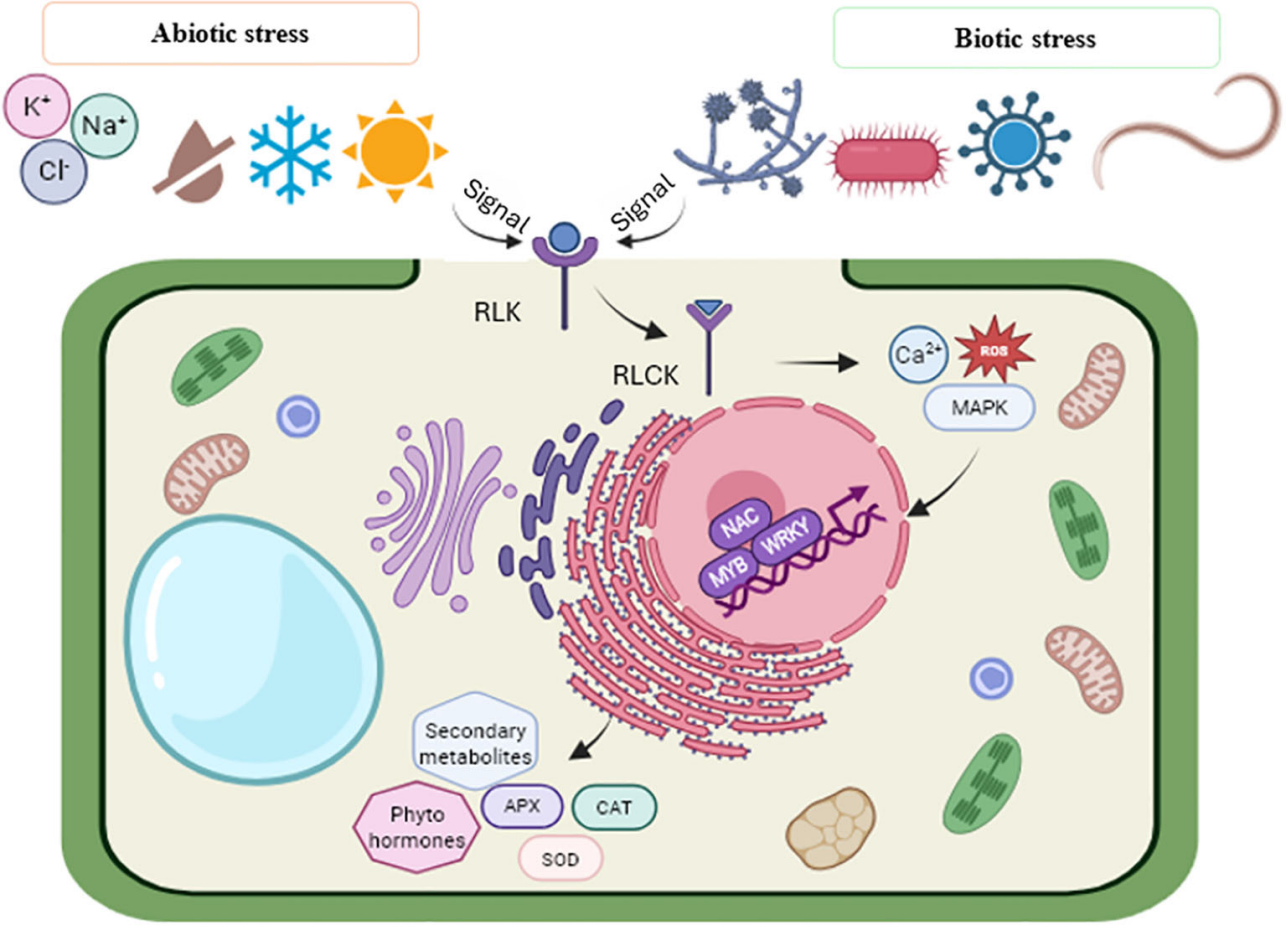
Schematic representation of the molecular reprogramming involved in secondary metabolite production during biotic and abiotic stress. In response to external stress signals, receptor-like kinases (RLKs) activate receptor-like cytoplasmic kinases (RLCKs), leading to increased calcium influx, ubiquitination and MAPK pathway activation. This triggers the activation of transcription factors such as NAC, MYB and WRKY. These molecular events initiate a series of biochemical responses, activating specific metabolic pathways and the production of ROS-scavenging enzymes such as CAT (catalase), GPX (glutathione peroxidase) and SOD (superoxide dismutase). It also promotes the synthesis of secondary metabolites and defense-related phytohormones to prevent infection. (Modified from Serag et al., 2023; Kumar et al., 2023). Created via Biorender.com.

Furthermore, it involves the synthesis of particular defense-related proteins, including defensins, chitinases and protease inhibitors. These molecular responses trigger a cascade of biochemical responses, leading to the activation of specific metabolic pathways and the synthesis of secondary metabolites to prevent further infection (**Figure 3**) (Serag et al., 2023; Jin et al., 2024; Shiade et al., 2024). For example, the major metabolic pathways activated during pathogen attack include the salicylic acid, alkaloid, jasmonic acid, and phenylpropanoid (Upadhyay et al., 2025). In response to biotic stress, plants synthesize phytoalexins, which are secondary metabolites such as terpenoids, phenols, alkaloids, and flavonoids, which posses defensive properties (Sharma et al., 2022). In contrast, phytoanticipants such as glucosinolates, cyanogenic glycosides and saponins are defense compounds that accumulate in plant tissues prior to pathogen exposure (Ishihara, 2021). Furthermore, an increase in chemodiversity has been demonstrated to increase protection against herbivore attack (Ziaja and Müller, 2023). The collective activation and regulation of these metabolic pathways in response to external stimuli emphasize the importance of chemodiversity as an adaptive trait that fosters plant survival under biotic stress.

### 4.3 Chemodiversity against biotic stress in eggplant

Eggplants are highly susceptible to various biotic stresses that significantly impact yield and quality (Talukder et al., 2025). *Leucinodes orbonalis* (shoot and fruit borer), *Bemisia tabaci* (whiteflies), *Aphis gossypii* (aphids), *Amrasca biguttula biguttula* (jassids), *Epilachna* spp. (hadda beetles), and *Tetranychus urticae* (red spider mites) are some of the insect pests that attack eggplant, causing considerable reductions in growth and yield. The larvae of *L. orbonalis* penetrate and grow into the shoots and fruits of their host, resulting in wilting and internal fruit damage. Aphids, jassids, and red spider mites suck cell sap, resulting in stunted plant growth and yield reduction (Shivalingaswamy et al., 2022). The most prominent eggplant pathogens are *Ralstonia solanacearum*, *Verticillium dahliae*, *Fusarium oxysporum*, *Botrytis cinerea*, *Alternaria solani* and *Pythium aphanideramatum*. These pathogens attack vascular tissues and aerial parts, leading to wilting, necrosis, and fruit rot (Upamanya et al., 2020; Kaniyassery et al., 2024b). The root knot nematode (*Meloidogyne incognita*) is a prevalent sedentary endoparasite of eggplant that feeds on roots and causes up to 60% loss in yield (Nyangwire et al., 2024).

When exposed to biotic stress, plants elicit signals that trigger proteins responsible for the activation of pathways regulating the biosynthesis of phytohormones, including auxins, ethylene, and brassinosteroids (Chen et al., 2021). Furthermore, Xiao et al. (2023) reported the activation of genes involved in the jasmonic acid and salicylic acid pathways, both of which are essential for PTI and play key roles in defense against biotic stress. Jasmonic acid is involved in regulating responses to wounding and is known as an inducer of systemic resistance, whereas salicylic acid is involved in the pathogen-induced defense pathway and acts as a signaling molecule to trigger systemic acquired resistance (SAR) in plants. These pathways form an integrated defense system that enables plants to perceive stress signals and elicit appropriate molecular and physiological responses (Majeed et al., 2024).

#### 4.3.2 Chemodiversity during resistance to pathogens

##### 4.3.2.1 Fungi

Fungal infection has been demonstrated to trigger significant variation in the eggplant chemical profile, thereby enhancing chemodiversity by influencing both primary and secondary metabolite production. For example, infection by *A. solani* has been reported to cause significant reductions in primary metabolites, such as carbohydrates and soluble proteins. Moreover, increase in the total phenolic content and free proline content were also observed (Attia et al., 2022). Proline acts as an osmoprotectant, thereby helping to stabilize proteins and membranes, in addition to scavenging ROS.

The impact of four fungal pathogens, namely, *Alternaria alternata*, *Fusarium solani*, *A. solani* and *Lasiodiplodia theobromae*, on the Mattu Gulla variety of eggplant were investigated by Kaniyassery et al. (2024a). As the infection progressed, a noticeable decline in the chlorophyll a content and net photosynthetic rate was observed. Moreover, the activity of antioxidant enzymes such as GPX, CAT, SOD, and ascorbate peroxidase (APX) were found to be increased, thereby facilitating the detoxification of ROS and maintaining cellular integrity. Metabolic pathway analysis revealed that, within one day of infection, several defense-related pathways involved in diterpenoid biosynthesis, sulfur and methionine metabolism, carotenoid biosynthesis, and glutathione metabolism were upregulated. By the seventh day, additional pathways regulating carotenoid biosynthesis, porphyrin and biotin metabolism were also activated. In the course of a 24-hour period of infection, the presence of *A. solani* and *A. alternata* triggered an increase in sesquiterpenoids and SGA, whereas infection with *F. solani* and *L. theobromae* resulted in the production of triterpenoids. After 7 days of infection, *A. solani* and *A. alternata* induced the synthesis of terpene glycosides and methylated flavonoids, whereas *F. solani* and *L. theobromae* continued to increase triterpenoid levels.

Transcriptomic and metabolomic profiling of eggplant infected with the fungus *V. dahliae* revealed significant upregulation of metabolic pathways associated with lysine degradation; flavone and flavonol biosynthesis; and tryptophan, cysteine and methionine metabolism. In addition, key defense-related proteins, including pathogenesis-related (PR) proteins, chitinases, peroxidases and β-1,3-glucanases were increased (Li et al., 2024b). The wild eggplant species *Solanum sisymbriifolium* was found to possess unique metabolites and molecular responses, thus demonstrating strong and chemically diverse defense mechanisms against fungal infections. Proteins involved in the phenylpropanoid and oxidative stress pathways were found to be upregulated during fungal infection (Wu et al., 2023).

##### 4.3.2.2 Bacteria

*R. solanacearum*, a soil borne bacteria causing bacterial wilt, poses a serious threat to eggplant, often leading to significant yield losses. In *R. solanacearum*-infected eggplant, increased biosynthesis of phenylpropanoids, flavonoids, zeatin, sesquiterpenoids, triterpenoids, cutin, suberin, wax and carotenoids has been observed (Yu et al., 2024). Xiao et al. (2023) reported that the levels of 1-O-Salicyl-D-glucose, (-)-jasmonoyl-L-isoleucine and 5′-glucosyloxyjasmonic acid significantly increased in eggplant following infection with *R. solanacearum*. Furthermore, pathways for the biosynthesis of phenylpropanoids, flavones, flavonols and phytohormones were upregulated.

##### 4.3.2.3 Virus

Viral infections in eggplant are caused by a number of viruses, including cucumber mosaic virus, tomato leaf curl virus, eggplant mild leaf mottle virus and alfalfa mosaic virus (Sofy et al., 2021; Mishra et al., 2023). Despite the clear impact of these viruses on eggplant health and productivity, studies investigating metabolite-level responses to viral infection in eggplant are limited.

A study conducted by Sofy et al. (2021) investigated the effect of alfalfa mosaic virus infection on eggplants. Photosynthetic pigments (carotenoids, chlorophyll a, chlorophyll b and total chlorophyll) were found to significantly decrease upon infection. Furthermore, alfalfa mosaic virus infection increased malondialdehyde (MDA) levels and the levels of ROS, such as hydroxyl radicals, superoxide ions, and hydrogen peroxide. There was also a significant upregulation of defense-related enzymes such as CAT, POD and SOD. Eggplant infected with alfalfa mosaic virus also presented an increase in flavonoids, phenols, lignin and endogenous salicylic acid.

##### 4.3.2.4 Nematodes

*M. incognita*, a type of root-knot nematode, has been identified as a significant cause of damage to root tissues, with the ability to compromise nitrogen uptake and overall plant vigour. This parasitic nematode poses a particular threat to eggplants. Recent research has investigated the genetic, biochemical, and microbiological factors behind nematode resistance in cultivated eggplant and its wild relatives in response to this significant threat. Transcriptomic studies of the roots of resistant eggplant infected with *M. incognita* revealed that the resistant plants activated a wide range of defense mechanisms, including increased phytohormone signaling components (jasmonic acid and salicylic acid) and pathogenesis-related enzymes such as PODs and glucanases. These responses have shown to facilitate cell wall fortification, increase the production of ROS, and lead to a localized hypersensitivity reaction that restricts the spread of infection (Zhang et al., 2021). Furthermore, infection of eggplant with *Meloidogyne javanica* increased the content of defense-related antioxidant enzymes such as POD, PPO, and phenylalanine ammonia lyase (PAL) (El-Shafeey et al., 2021).

##### 4.3.2.5 Insects

Eggplants accumulate high concentrations of monoterpenes, including compounds such as limonene and geraniol. These compounds can function either directly or indirectly in defense, as signaling molecules or as insect repellents. Similarly, geraniol was found to be a strong oviposition deterrent. In a study on the chemical ecology involved in the antixenosis of Himalayan eggplant varieties, geraniol was found to be a strong oviposition deterrent against *L. orbonalis*. The results obtained demonstrate the role of certain volatiles in facilitating antixenosis, a resistance mechanism in which the plants use chemical cues to steer the pests away from it. Resistant eggplant genotypes presented relatively high levels of total phenols, PPO, POD, PAL, and solasodine in fruit and shoot tissues, whereas susceptible genotypes presented reduced levels of these biochemical characteristics (Challa et al., 2021). Upon infestation by *Tuta absoluta*, eggplant maintained relatively high levels of amino acids, sugars (fructose and sucrose), salicylic acid, jasmonic acid, and total phenols, particularly at 48 hours post‐infestation (Chen et al., 2021) (**Table 1**).

**Table 1:** Effects of biotic stress on eggplant physiology, tolerance mechanisms and management measures.

Causative organism		Biochemical responses involved in tolerance mechanisms	Functional role of biochemical responses	References
Fungi	A.solani	Increased total phenolic content and proline.	Induced systemic resistance in host osmolyte and ROS scavenger.	Attia et al., 2022
	*V. dahliae*	Enhanced flavone and flavonol biosynthesis. Improved tryptophan, cysteine and methionine metabolism Improved chitinase, POD and β-1,3-glucanase activity	Facilitated the mitigation of oxidative damage	Wu et al., 2023
	*A.solani* and *A. alternata*	Increased antioxidant enzyme activity- SOD, GPX, CAT, APX. Elevated sesquiterpenoids and steroidal glycosides content. Improved the content of terpene glycosides and methylated flavonoids.	Defense against ROS accumulation. Acted as phytoalexins and phytoanticipants	Kaniyassery et al. (2024a)
	*F. solani* and *L. theobromae*	Enhanced antioxidant enzyme activity- SOD, GPX, CAT, APX. Boosted triterpenoids content.	Played key role in ROS scavenging.	
Bacteria	*R. solanacearum*	Upregulated salicylic acid and jasmonic acid pathway.	Induced systemic resistance and triggered SAR.	Xiao et al., 2023
		Enhanced phenylpropanoid, flavonoid, zeatin, sesquiterpenoid and triterpenoid.	Induces resistance to bacterial wilt.	Yu et al., 2024
Virus	Alfalfa Mosaic Virus	Increased MDA, SOD, POD, CAT and endogenous salicylic acid.	ROS scavenging and induced SAR.	Sofy et al., 2021
Nematode	*M. incognita*	Enhanced phytohormone (salicylic acid and jasmonic acid) signaling components. Increased POD and glucanase activity.	Triggered systemic resistance and SAR Mitigation of oxidative damage.	Zhang et al., 2021
	*M. javanica*	Improved PPO, POD and PAL activity.	Improves tolerance towards oxidative stress PAL plays crucial role in phenylpropanoid metabolism	El-Shafeey et al., 2021
Insects	*L. orbonalis*	Elevated concentration of PPO, POX, PAL and solasodine	Improved tolerance towards oxidative stress	Challa et al., 2021
	*T. absoluta*	Increased salicylic acid, jasmonic acid and total phenol content.	Triggered induced systemic resistance and SAR	Chen et al., 2021

### 4.4 Chemodiversity in response to abiotic stress in eggplant

Abiotic factors such as sunlight, flooding, temperature, salinity, and drought have been shown to induce stress in plants, thereby altering their normal growth, development and biochemical composition (**Table 2**). Genetic, physiological, biochemical and molecular factors play key roles in helping plants survive adverse environmental conditions. The analysis of these factors is crucial for the development of new breeding lines that are capable of rapidly recovering from stress caused by abiotic factors (Toppino et al., 2022).

**Table 2:** Effects of abiotic stress on the physiology and tolerance mechanisms of eggplants.

Stress type	Cultivar/variety/accession/genotype			Biochemical responses involved in tolerance mechanisms	Functional role of biochemical responses	References
Salinity	Moderately susceptible		L-888	Decreased K^+^ and Ca^2+^ Higher Na^+^ in shoot	–	Shahbaz et al., 2013
	Susceptible		Round	Decreased K^+^ and Ca^2+^ Higher Na^+^ in root		
	Tolerant		Bonica and Galine	Increased proline, MDA. Accumulation of Na^+^	Osmoprotectant, suggested cellular membrane damage	Hannachi and Van Labeke, 2018
	Susceptible		Adriatica and Black Beauty	Increased proline. Reduction in level of Ca^2+^, Mg^2+^ and K^+^	Osmoprotectant	
	Tolerant		HBR-314-E, ICS-BR-1351, HBR-334-D, and HBR-313-D	Enhanced CAT, PO and SOD, MDA activity	Improved tolerance towards oxidative stress	Jameel et al., 2024
	Tolerant		Poluru Vanga, Bhagyamathi, Manapparai, Pusa Hybrid-6, Arka Harshitha, IIHR-322, IIHR-633, IIHR-832, IIHR-766, IIHR-766-A	Increased CAT, POD, GR, APX and PPO activity	Enhanced tolerance towards oxidative stress	Harsha et al., 2025
	Susceptible		*S. gilo*, *S. macrocarpon*, Kanta Baigan, Pusa Kranti, Sharapova Bottle Brinjal, IIHR-3, Utkal GR, Utkal Anushree, Punjab Barsati	Elevated MDA levels	Indicated oxidative stress and cellular membrane degradation	
	Tolerant		FLT E635, MM00007, MM01010	CAT, POD and SOD relatively high Protein, glycine betaine increased	Alleviated oxidative stress and facilitates osmotic adjustment	Yousefi et al., 2025
	Susceptible		FLT10, FLT22, FLT46, FLT E9012, M-AK, MM00197, MM00960	Protein, glycine betaine content were improved		
Drought	Tolerant		Arka Harshitha	Elevated levels of MDA, SOD and POD Higher levels of mannose, fructose, glucose, inositol, fucose, ribose and arabinose	Mitigated oxidative stress and implied cellular membrane degradation	Harsha et al., 2024
	Susceptible		*S. melongena* var. *insanum*	Boosted the level of glucose, manoose, fructose, sucrose, xylose and arabinose	Protect membranes and proteins from stress damage and helps in osmotic adjustment	
	Tolerant		Bonica and Galine	Increased proline content, SOD, CAT and APX. High levels of N, P and K	Osmoprotectant, and alleviated free radical scavenging	Hannachi et al., 2022
	Susceptible		Adriatica and Black Beauty	Increased MDA level, proline	Suggested cellular membrane degradation Functioned as osmoprotectants	
Temperature	Heat	*S. melongena* cv. Huqie 9		Improved MDA, SOD, POD, CAT and APX activity	Facilitated tolerance towards oxidative stress	Wu et al., 2014
		Tolerant	Tewangda	Increased anthocyanin content	Free radical scavengers	Zhang et al., 2019
		Tolerant	Bonica and Galine	Enhanced proline, salicylic acid, abscisic acid and jasmonic acid content SOD, CAT and APX activity increased	Functioned as osmoprotectants. Triggered induced systemic resistance and SAR ROS scavenging	Hannachi et al., 2022
		Susceptible	Adriatica and Black Beauty	Increased levels of MDA and jasmonic acid	Implied cellular membrane degradation. Induced systemic resistance.	
	Cold	*S. melongena* cv. Hadrian		Elevated electrolyte leakage and MDA. SOD, CAT and POD activity decreased	Indicated cellular membrane degradation. Functioned as ROS scavenger.	Barzegar et al., 2021
		*S. melongena* var. Xiuniang		Upregulation of flavonoid, phenylpropanoids and anthocyanins	Alleviated oxidative damage	Shen et al., 2025

#### 4.4.1 Salinity stress

One of the most prevalent and major threats to agriculture is salinity stress. Currently, around 1.38 million hectares of land, or 10.7% of the planet’s surface area, are affected by salinity (FAO, 2024, https://www.fao.org/family-farming/detail/en/c/1730916/). Approximately 6.74 million hectares of land in India are affected by this stress, and estimates show that the salinization of farmed land is expected to rise by 10% per year (Harsha et al., 2025). Salinity can induce osmotic damage, nutritional imbalance, and downregulation of enzyme activity in plants. Regular plant function is negatively impacted by salinity-induced oxidative stress. It damages proteins, nucleic acids, and cellular membranes and decreases their photosynthetic efficiency, leading to poor development (Jameel et al., 2024).

A plethora of adaptation strategies to counteract salinity stress have been thoroughly investigated in various plant species. In the context of salinity stress, numerous eggplant genotypes exhibit salt tolerance, characterized by elevated levels of antioxidant enzyme activity and optimal physiological health. Owing to their negative effects on stomatal conductance and membrane integrity, elevated levels of Cl^-^ and Na^+^ in leaves are positively correlated with increased canopy temperature and MDA content. To improve ion homeostasis via osmotic balance, tolerant genotypes have been shown to limit the uptake of Na^+^ and Cl^−^ while preserving optimal levels of K^+^, Ca^2+^, and Mg^2+^ (Harsha et al., 2025). Chloroplasts, together with other organelles such as mitochondria and peroxisomes, are the main sources of ROS; hence, when excessive amounts of ROS are produced, photosynthetic pigments such as carotenoids, chlorophylls, and photosystems are unable to function as intended (Dorga and Kim, 2020).

Salinity stress has been reported to significantly increase Na^+^ accumulation in both shoot and root tissues of 2 eggplant cultivars (L-888 and Round), with L-888 exhibiting a greater Na^+^ accumulation in shoots and the Round cultivar exhibiting an increased accumulation of Na^+^ in roots. Furthermore, the K^+^ and Ca²^+^ levels also decreased in both cultivars under salinity stress, especially in the shoots. Moreover, the net CO_2_ assimilation rate (A), transpiration rate (E), and water use efficiency (A/E, which indicates how efficiently a plant can use water to fix carbon during photosynthesis) decreased significantly under salinity stress. Furthermore, photosystem II efficiency (Fv/Fm), which represents the maximum efficiency of photosystem II during the light reactions of photosynthesis, decreases with increasing salinity, indicating photoinhibition (Shahbaz et al., 2013).

Hannachi and Van Labeke (2018) studied the salinity tolerance and physiological alterations of four distinct eggplant cultivars: Black Beauty, Bonica, Adriatica, and Galine. The soluble carbohydrate levels increased in the Black Beauty and Adriatica cultivars, whereas Galine and Bonica presented starch accumulation and reduced soluble sugars. A significant increase in proline levels was detected in all the cultivars, with maximum increase observed in the Black Beauty and Adriatica cultivars. Compared with those in tolerant cultivars, MDA levels in sensitive cultivars increased 10–11-fold, whereas MDA levels in tolerant cultivars increased 2–3-fold. Salinity stress also has considerable effects on the mid-day leaf osmotic potential (ψπ) and leaf water potential (ψl), which are key indicators of a plant’s water status and ability to maintain turgor under saline conditions. In susceptible cultivars, the leaf osmotic potential (ψπ) and leaf water potential (ψl) significantly decrease, indicating impaired water uptake and turgor loss. Tolerant cultivars (Bonica and Galine) maintained stable Ψl and Ψπ even at high salinity. Salt stress caused a reduction in the levels of Ca²^+^, Mg²^+^ and K^+^ in the leaves of susceptible cultivars (Adriatica and Black Beauty). The Adriatica and Black Beauty cultivars accumulated significantly greater concentrations of Na^+^ in their leaves and were unable to maintain normal growth levels under salinity stress, whereas Bonica and Galine presented low Na^+^ accumulation.

Brenes et al. (2020) reported the biochemical and physiological responses of eggplant and its wild relative *Solanum torvum* to salinity stress. In both plant species, salinity had no effect on the specific activity of antioxidant enzymes, the synthesis of antioxidant molecules, or the activation of antioxidant systems. Both species accumulated Na^+^ and Cl^-^ in roots and leaves with increasing salinity. Eggplant retained more ions in its roots, whereas *S. torvum* actively transported them to its leaves. In addition, K^+^ levels remained stable in eggplant but fluctuated in *S. torvum*. Furthermore, the MDA content increased only in eggplant.

Four eggplant genotypes (HBR-314-E, ICS-BR-1351, HBR-334-D, and HBR-313-D) were chosen to assess the impact of salt stress (Jameel et al., 2024). The results revealed that the chlorophyll content significantly decreased as the carotenoid content increased. These findings indicate that an adaptive reaction to high salinity-induced oxidative damage is essential in every plant. Furthermore, the total protein content and flavonoid content increased significantly, whereas the content of total soluble sugars sharply decreased. Antioxidant enzymes, including CAT, PO, and SOD, become active under salt stress, which helps prevent oxidative damage. Additionally, the MDA and hydrogen peroxide (H_2_O_2_) levels increased in all the varieties.

Harsha et al. (2025) screened ten highly tolerant (Bhagyamathi, Poluru Vanga, Manapparai, Arka Harshitha, Pusa Hybrid-6, IIHR-766, IIHR-766-A, IIHR-322, IIHR-633, IIHR-832) and nine highly susceptible (*S. gilo*, *S. macrocarpon*, Pusa Kranti, Kanta Baigan, Sharapova IIHR-3, Bottle Brinjal, Utkal Anushree, Utkal GR, Punjab Barsati) genotypes of eggplant for salinity stress. The response to salinity stress is dependent on the genotype. Chlorophyll degradation was more significant in susceptible genotypes, whereas tolerant genotypes did not exhibit pigment degradation. Na^+^ and Cl^-^ accumulation is high in the roots and leaves of susceptible varieties, disrupting osmotic balance and metabolic function. Furthermore, susceptible genotypes also presented increased MDA contents, revealing severe lipid peroxidation and membrane damage, whereas tolerant genotypes presented lower MDA levels, suggesting effective ROS scavenging. In addition, the enzymatic activities of key antioxidant enzymes, such as CAT, glutathione reductase (GR), APX, PPO and POD were greater in the tolerant genotypes and were able to mitigate oxidative damage during stress.

Yousefi et al. (2025) studied the effects of salinity stress on ten eggplant accessions [FLT10 (V1), FLT22 (V2), FLT46 (V3), FLT E635 (V4), FLT E9012 (V5), M-AK (V6), MM00007 (V7), MM00197 (V8), MM00960 (V9), and MM01010 (V10)]. The results revealed that accessions V4, V7, and V10 presented low-level electrolyte leakage (efflux of electrolytes from cells) and increased cell membrane stability in their leaf cells, indicating that they were more tolerant to salinity stress. Glycine betaine, total protein, and antioxidant defense enzymes such as POD, CAT and SOD were relatively high in the leaves of accessions V4, V7, V8, and V10 but relatively low in those of accessions V1, V2, V3, V5, V6, and V9. The increased prevalence of these traits in the first group indicates that these accessions have a better defense mechanism under salt stress. Furthermore, the total protein content and glycine betaine, an osmoprotectant, increased in all the accessions under salinity stress.

#### 4.4.2 Drought stress

Drought stress is one of the most important factors affecting agricultural production worldwide. The growth and productivity of eggplant are adversely affected by drought stress. In eggplant, increased drought led to a sequential decrease in yield with a reduction in plant height, days for flower initiation, fruit length, average fruit weight, circumference, and width. However, in some cases, exposure to drought stress results in an increase in the number of fruits and branches, indicating tolerance to drought (Faizan et al., 2021).

The physiological, morphological, and metabolomic responses to drought stress were evaluated in the drought-sensitive *Solanum melongena* var. *insanum* and the drought-tolerant *Solanum melongena* var. Arka Harshitha at three different intervals. Compared with the sensitive genotype, the tolerant genotype Arka Harshitha presented decreased lipid peroxidation and increased antioxidant enzyme activity (SOD and POD), suggesting enhanced ROS scavenging capacity. In addition, under both the control and drought conditions, genotype-specific patterns of sugar accumulation were identified in the leaves and roots. The tolerant genotype Arka Harshitha presented relatively high levels of mannose, fructose, glucose, inositol, fucose and sucrose. In contrast, carbohydrates, mannose, xylose, sucrose, arabinose, fructose, and glucose accumulated in the sensitive genotype *Solanum melongena* var. *insanum*. Ribose and arabinose showed genotype-dependent accumulation, with ribose being significantly enriched in Arka Harshitha leaves under stress. Relatively high concentrations of these sugars positively controlled osmotic balance, energy metabolism, and signaling during drought (Harsha et al., 2024).

The effects of drought stress on the biochemical and physiological parameters of four eggplant cultivars, Galine, Bonica, Adriatica and Black Beauty, were evaluated. These findings revealed that drought resulted in increased H_2_O_2_ accumulation, proline content, and MDA concentration. The levels of H_2_O_2_ significantly increased in Bonica and Galine, and the increase in MDA was greater in Adriatica and Black Beauty than in Bonica and Galine. This suggests the presence of greater oxidative damage in Adraitca and Black Beauty. Furthermore, an increase in proline content was observed in all four genotypes of eggplant tested, with the highest levels recorded in Bonica and Galine. These findings indicate that these cultivars rely more strongly on osmotic balance through proline accumulation. Additionally, the relative water content, which is indicative of the amount of water lost through transpiration and the amount transported to the leaf tissues, substantially decreased in all four cultivars. The activities of defense enzymes such as CAT, SOD and APX were three-fold greater in Bonica and Galine, indicating greater ROS detoxification. Moreover, an elevated level of the stress hormone abscisic acid was observed in response to drought conditions, which improved stomatal regulation thereby enhancing stress adaptation. All eggplant cultivars presented a decrease in nutrient uptake; however, Adriatica and Black Beauty presented lower root, stem, and leaf K, P, and N levels than did Galine and Bonica, suggesting a weaker capacity for nutrient accumulation (Hannachi et al., 2022).

Leaf samples of 19 eggplant accessions exposed to drought stress were analysed via GC-MS, and 14 organic acids, 8 sugars, and 7 amino acids were identified. After a period of four weeks, during which the plants were subjected to conditions of drought stress, the plants presented the most profound metabolic divergence, especially in the tolerant accessions. Significant accumulations of sucrose, fructose, trehalose, mannose, and xylose under drought stress were recorded. These sugars serve as osmoprotectants and energy reserves, and their accumulation is correlated with delayed wilting and increased drought tolerance. Significant increase in proline and glutamate contents were also observed. Proline and glutamate play pivotal role in osmotic adjustment as well as in ROS scavenging. Furthermore, citric acid, fumaric acid, malic acid, and isocitric acid levels increased, suggesting increased respiration. They also facilitate the biosynthesis of secondary metabolites that are important for defense against stress (Mibei et al., 2018).

#### 4.4.3 Temperature stress

High temperatures caused by changing climatic conditions can damage eggplant. In response to temperature changes, especially heat stress, eggplant has exhibited chemodiversity, with significant changes in gene expression and phytochemical profiles. According to transcriptome profiling studies, heat stress leads to the overexpression of genes involved in stress responses, such as heat shock proteins and ROS detoxification enzymes, as well as those governing secondary metabolism (Zhang et al., 2020).

The exposure of *S. melongena* cv. Huqie 9 to elevated temperatures (43/38 °C) resulted in various physiological changes, including photosynthetic inhibition and oxidative stress. Significant decrease in chlorophyll content, stomatal conductance, and PSII efficiency were observed along with elevated levels of ROS. This resulted in the increase of lipid peroxidation, suggesting membrane damage. Antioxidants enzymes such as POD, SOD, APX and CAT showed an increased activity when exposed to heat stress. Furthermore, increase in proline, soluble sugar and protein accumulation were observed under stress (Wu et al., 2014).

In a study conducted by Zhang et al. (2019), a heat-resistant eggplant cultivar (‘Tewangda’) was exposed to temperatures of 38 °C and 45 °C for 3 and 6 hours, respectively. The results indicated a substantial decline in anthocyanin content with increasing temperature and duration of exposure. A minimal decrease in concentration was observed following short-term exposure of 3 hours; however, biochemical assays confirmed a progressive decrease in anthocyanin concentration, particularly at 45 °C for 6 hours.

Hannachi et al. (2022) investigated the effects of heat stress on four eggplant cultivars (Adriatca, Black Beauty, Bonica and Galine). Heat stress significantly decreased carotenoids, chlorophyll a, chlorophyll b and total chlorophyll in susceptible cultivars (Black Beauty and Adriatica). Increased concentrations of H_2_O_2_ and MDA were also recorded. The cultivars Galine and Bonica presented significant accumulation of proline under heat stress. In addition, increase in SOD, CAT and APX activities were also observed. While heat stress increased the production of defense hormones like abscisic acid and salicylic acid in all the cultivars, the jasmonic acid concentration increased only in the tolerant cultivars.

Additionally, low-temperature exposure is reported to affect eggplant productivity. Barzegar et al. (2021) investigated the effect of low temperature on eggplant (*S. melongena* cv. Hadrian) fruits after harvest by storing them at 4 °C for 12 days. Elevated electrolyte leakage and MDA levels were observed along with a decrease in the total phenolic and flavonoid contents. The activity of antioxidant enzymes such as SOD, POD and CAT was also reduced under low-temperature stress. According to a study conducted by Shen et al. (2025), anthocyanin accumulation increases significantly in the peel, stem epidermis, petals, and calyx of *S. melongena* var. Xiuniang upon exposure to low temperatures. In addition, pathways involved in the biosynthesis of flavonoids, phenylpropanoids and anthocyanins were upregulated.

### 4.5 Mitigation strategies for abiotic and biotic stresses

To ensure sustainable production, it is essential to develop and implement effective mitigation strategies against these stresses. The application of biostimulants and stress-tolerant microbes for mitigating stress in eggplant has been documented.

The utilization of microbial treatments is becoming prevalent as an environmental friendly alternative to genetic engineering techniques. A recent study used plant growth-promoting rhizobacteria (PGPR) to restore nematode-susceptible soil biota. The application of beneficial microorganisms such as Bacillus and Pseudomonas has been shown to increase plant's resistance to *M. incognita* and promote plant development by increasing antioxidant enzyme activity and triggering induced systemic resistance (ISR). This strategy promoted the application of microbial engineering as a sustainable way to regulate infestation by biological nematodes (Ali et al., 2024). Eggplant resistance to root-knot nematodes is the result of a complex interaction between defense activation aided by the microbiome, chemodiversity-driven responses, and genetic resistance mechanisms from wild relatives. These results pave way for integrated biocontrol and breeding approaches through improved rhizospheres and favourable host genetics.

Two strains, *Pseudomonas stutzeri (*KS5C) and *Bacillus tequilensis* (KS5B), were assessed for their impact on eggplant under drought stress conditions on the basis of their plant growth-promoting properties and osmotic stress tolerance. The shoot and root lengths of eggplants inoculated with osmotic stress-tolerant bacteria significantly increased. In addition, the inoculated plants outperformed the control in terms of antioxidant activity, such as peroxidase, and PPO activity, proline, and chlorophyll content (Patel et al., 2022).

The effects of exogenous treatment with proline as a foliar spray on chlorophyll fluorescence, mineral ion buildup, gas exchange properties and growth in the two eggplant cultivars Round and L-888 bred under saline conditions were examined. Exogenous proline treatment alleviated the negative impact of salt stress on shoot fresh weight in both eggplant cultivars but improved the A/E ratio in the Round cultivar only (Shahbaz et al., 2013).

## 5 Analytical approaches for studying eggplant chemodiversity

Metabolomics is a crucial tool in plant breeding, as it helps to identify metabolic markers associated with the response of plants to stress, aiding in crop improvement (Razzaq et al., 2022). Eggplant metabolites have been characterized, and attempts have been made to correlate these profiles with nutrient use efficiency, fruit morphology, response to biotic and abiotic stress, and other desirable traits (Saha et al., 2023). Through these studies, more than a thousand compounds have been detected in different eggplant cultivars. To further explore and interpret this chemical diversity, contemporary research now employs advanced analytical platforms with metabolomics.

Metabolomics consists of a range of high-throughput technologies, like gas chromatography–mass spectrometry (GC-MS), liquid chromatography–mass spectrometry (LC-MS) and nuclear magnetic resonance (NMR) spectroscopy each offering unique advantages in sensitivity, resolution, and metabolite coverage. LC-MS analysis is a widely used technique because of its ability to detect a broad spectrum of metabolites, such as phenolic acids, flavonoids, alkaloids and terpenoids (Chauhan et al., 2024). Sample preparation techniques, extraction protocols, and instrument parameters are optimized to maximize metabolite yield and reproducibility, enabling both targeted quantification of known compounds and untargeted discovery of novel metabolites.

Integration of genetic tools such as high-throughput sequencing with metabolomics has been efficiently employed to understand the genetic factors controlling secondary metabolite pathways in eggplant (Wang et al., 2024). High-throughput sequencing is a technology that enables the simultaneous sequencing of several DNA molecules (Hu et al., 2021). This technology facilitates rapid analysis of the genetic and molecular basis of plant secondary metabolite biosynthesis (Dasanya and Hewadikaram, 2024). Recent studies have integrated high-throughput sequencing, particularly RNA-Seq, with metabolomic profiling to investigate secondary metabolite biosynthesis in eggplant. Furthermore, transcript–metabolite network analyses, like Weighted Gene Co-expression Network Analysis (WGCNA), has facilitated the elucidation of the mechanisms by which genetic regulators control these metabolites under stress conditions, including drought, waterlogging, salinity, heat, and pathogen attacks.

The datasets generated by these techniques are typically large and complex, necessitating the use of sophisticated bioinformatics tools for accurate data preprocessing, including peak detection, deconvolution, alignment, and normalization. Software such as XCMS, MZmine, MS-DIAL, and MetaboAnalyst facilitates these steps and provides robust statistical and multivariate analysis frameworks. This aids in the identification of differentially accumulated metabolites and metabolic signatures linked to genotype, stress conditions, and postharvest treatments (Liu et al., 2021; Niu et al., 2022).

Additionally, metabolite annotation and structural elucidation have significantly improved through the use of comprehensive key databases, including METLIN, MassBank, PMN and KEGG (Yi and Zhu, 2020) (**Table 3**). These data repositories provide crucial biochemical context by linking metabolites to metabolic pathways and enabling cross-species comparisons. Integration of metabolomic data with proteomic, transcriptomic, and genomic datasets through bioinformatics pipelines facilitated the understanding of metabolic regulation in eggplant, highlighting key genes and enzymes regulating biosynthetic pathways (Banerjee et al., 2021; Gerasimoska et al., 2021; Giera et al., 2024).

**Table 3:** Overview of metabolic databases with their applications and access to URLs.

Database	Application	URL
XCMS (eXtensible Computational Mass Spectrometry)	Analysing metabolic pathways using raw metabolomic data, with integration of proteomic and genomic information.	https://xcmsonline.scripps.edu/landing_page.php?pgcontent=mainPage
Mzmine	Processing and visualizing mass spectrometry data.	https://mzmine.github.io/mzmine_documentation/
MS-DIAL (Mass Spectrometry–Data Independent AnaLysis)	Untargeted molecular data collection. Simultaneous identification and quantification of small molecules.	https://systemsomicslab.github.io/compms/msdial/main.html
MetaboAnalyst	Comprehensive metabolomics data analysis. Interpretation and integration with other omics data.	https://www.metaboanalyst.ca/
METLIN (METabolite LINk)	Supports diverse metabolite research and helps identify metabolites using mass spectrometry data.	https://metlin-nl.scripps.edu/landing_page.php?pgcontent=mainPage
MassBank	Mass spectral library for identifying small molecules in metabolomics, exposomics, and environmental studies.	https://massbank.eu/MassBank/
KEGG (Kyoto Encyclopedia of Genes and Genomes)	Provides tools for browsing genome maps, comparing two genome maps, manipulating expression maps.	https://www.genome.jp/kegg/
PMN (Plant Metabolic Network)	Broad network of plant metabolic pathway databases with computational predictions of enzymes and pathways.	https://www.plantcyc.org/

A variety of analytical techniques that combine biochemical, genetic, and statistical methodologies have been reported for characterizing and distinguishing various eggplant accessions to study their chemodiversity in response to several stresses. Hierarchical Cluster Analysis (HCA) and Principal Component Analysis (PCA) are among the multivariate tools employed in the statistical analysis of biochemical diversity. These tools facilitate efficient categorization of genotypes according to their biochemical profile post analysis (Chanana et al., 2020). Therefore, integrating biochemical, molecular and statistical approaches facilitates the understanding of the synthesis and accumulation of secondary metabolites in crops through the combined use of metabolite profiling, gene expression analysis, and regulatory network mapping techniques.

## 6 Genetic regulation of chemodiversity by metabolic pathways

The underlying genetic and transgenic variation influences metabolites in plants, which is directly reflected in the complexity of their chemical profiles (Hayashi et al., 2023). The regulation and evolution of biosynthesis-related gene clusters (BGCs), which group functionally related genes involved in the same metabolic pathway, are key factors driving metabolic diversity (Pereira, 2024). BGCs involved in the synthesis of acylsugars and withanolide occur in eggplant and other Solanaceae members because of gene duplication, subfunctionalization, and chromatin-level control. They can occasionally display tissue-specific activity because of unique epigenetic changes. This enables a significant variety of metabolite profiles, efficient coregulation of pathways, and opportunities for adaptive evolution (Priego-Cubero et al., 2025; Smit and Lichman, 2022).

The biosynthesis of important metabolites such as flavonoids, lignins, and phenolic acids, which support fruit color, antioxidant activity, and pathogen defense, occurs through the phenylpropanoid pathway (Talukder et al., 2025). In eggplant, various gene families have evolved to encode enzymes such as PAL, cinnamate-4-hydroxylase (C4H), and 4-coumarate:CoA ligase (4CL). One of the main causes of chemodiversity among eggplant cultivars is differences in the structure and regulation of these pathways. Many eggplant fruits obtain their distinctive purple hue through the accumulation of different anthocyanins, which is controlled by the flavonoid biosynthesis pathway and coordinated by the MYB-bHLH-WD40 (MBW) transcriptional complex (Li et al., 2024a). According to genetic and comparative transcriptomic research, tissue specificity and pigment accumulation intensity are determined by the expression of structural genes such as *DFR*, *CHS*, *ANS*, and *CHI*, which are regulated primarily by transcription factors. Light and temperature are two examples of environmental variables that might further alter MBW activity and consequently influence the anthocyanin concentration (Manickam et al., 2025).

SGAs are signature defense metabolites, and their production is strictly regulated by the glycoalkaloid metabolism (GAME) gene cluster and a set of cytochrome P450 enzymes. In addition, SmJRE4, a jasmonate-responsive ERF transcription factor, directly regulates the biosynthesis of SGA in eggplant. The adaptability of the eggplant defense metabolome and sensitivity to environmental stimuli are highlighted by these transcriptional regulatory mechanisms (Shoji and Saito, 2022). Another example of specialized metabolism influenced by genetic regulation is the generation of acylsugars in eggplant. This is especially true for the *ASAT* gene family, which encodes the acyltransferase enzymes that are responsible for the variation in the composition of the acyl chain present in glandular trichomes (Fiesel et al., 2024). The *ASAT* family’s gene duplication, expansion, and substrate specificity support the plant’s defense against insect herbivores and pests, thus resulting in lineage-specific metabolic profiles. These processes demonstrate how chemodiversity and its adaptive advantages are supported by genetic advancements in metabolic pathways (Smit and Lichman, 2022).

Research using metabolic profiling, QTL mapping, and genomics has revealed the multigenic and quantitative characteristics of metabolite accumulation in eggplant (Zhou et al., 2023). The relationships among gene expression, enzyme function, and metabolite accumulation are becoming more clearly defined. Important metabolic QTLs impacting the concentrations of SGA (F-SOLM.6.1, F-MSOLM.6.1), anthocyanin (P-D3R.5.1, P-NAN.5.1), and glycosylated flavonol (P-RUT.10.1, P-KSOPH.10.1) have been mapped. These discoveries provide insights for breeding strategies using targeted gene editing of metabolic pathway genes to improve fruit attractiveness, resistance, and nutritional quality (Sulli et al., 2021).

## 7 Environmental and agronomic influences on metabolic pathway gene expression

Important metabolic genes, such as those governing pigment biosynthesis, antioxidant defense, osmolyte and secondary metabolite accumulation, are influenced by external stress. The signals produced by external stress activate transcription factors, which in turn upregulate genes involved in metabolite biosynthesis. Visible physiological reactions frequently mirror these transcriptional alterations (Liu et al., 2024). Transcriptomic profiling under heat stress conditions (38-45 °C) revealed the downregulation of several core genes of the anthocyanin biosynthesis pathway in eggplant. These genes included *CHI3, CYP75A2, PAL2*, *CHSB* and *ASAT*. Furthermore, the expression levels of the transcription factors PHL11 and bHLH35 were significantly elevated, indicating a temporary compensatory mechanism (Zhang et al., 2019). The Tewangda variety of eggplant presented differential expression of many genes, including those involved in trehalose phosphate synthase (*TPS*) activity and proline biosynthesis (*P5CS1*), in response to heat stress. Trehalose phosphate synthase and proline aid plants in adapting to heat stress by acting as osmoprotectants. Additionally, 57 genes associated with antioxidant defense, such as *GR*, *GPX*, *POD*, *APX*, and glutathione-S-transferase (*GST)*, were also differentially expressed, indicating a broad transcriptional response to oxidative and osmotic stress. These findings indicate broad-spectrum transcriptional and metabolic reprogramming to reduce oxidative damage (Zhang et al., 2020).

Cai et al. (2024) conducted RNA-seq on two varieties (Fuguhuajiao, which is cold tolerant, and Fengxiandahongpao, which is cold sensitive) of eggplants exposed to cold stress. A total of 7,024 genes were differentially expressed in Fuguhuajiao, whereas 6,209 genes were differentially expressed in Fengxiandahongpao. In Fuguhuajiao, genes involved in terpenoid biosynthesis (*SQS*, *CAS*, and *LAS*) and abscisic acid signaling (*PP2C and SnRK2*) were upregulated. In Fengxiandahongpao, genes regulating starch/sucrose metabolism (*HK*, *ISA*, *TreY*, *TreZ*, *AMY*) and chlorophyll degradation (*CLH*, *SGR1*, *SGR2*) were upregulated. WGCNA revealed that the key module MEgrep60 was increased in plants subjected to cold stress. Furthermore, the membrane transporter MDR1 and numerous transcription factors, such as MYB, AP2/ERF, bZIP, bHLH, and C2H2, were also upregulated.

A study conducted by Villanueva et al. (2023) on the effects of drought stress on eggplant and its related wild species, *Solanum dasyphyllum*, revealed differential expression of genes involved in α-linolenic acid metabolism and phenylpropanoid, flavonoid, and carotenoid biosynthesis. *PYRPYR/PYL/RCAR, PP2Cs*, *SnRK2s*, and AREB/ABF transcription factors governing abscisic acid synthesis were upregulated. Furthermore, genes involved in glutathione metabolism, photosynthesis, antenna protein, porphyrin and chlorophyll metabolism, and α-linolenic acid metabolism were differentially expressed. Pathways involved in the biosynthesis of zeatin, galactose, triterpenoid and sesquiterpenoid were also enriched.

It has been demonstrated that salt stress, another abiotic challenge, significantly alters eggplant metabolism. The AP2/ERF transcription factor SmERF1 was one of the 8,509 genes that were differentially expressed in eggplant roots treated with NaCl. SmERF1 positively regulates ABA-mediated signaling pathways, which mediate salt tolerance. Functional analyses revealed that silencing SmERF1 significantly decreased the activity of important antioxidant enzymes (CAT and SOD) and suppressed salinity stress defense-related genes such as *SmNCED1*, *SmNCED2* and *SmDHN1* (Shen et al., 2022). Under salinity stress, the MYB transcription factor SmMYB39 was overexpressed in the ML41 variety of eggplant. This facilitated the accumulation of ROS, soluble sugars, and proline. KEGG pathway analysis revealed that SmMYB39 is also involved in nicotinate, nicotinamide, alanine, aspartate, glutamate, and 2-oxocarboxylic acid metabolism. An analysis of the SmMYB39 promoter via the PlantCARE online tool revealed several stress-related regulatory elements, including sites that respond to abscisic acid, jasmonic acid, ethylene, and salicylic acid, and binding sites for other MYB and WRKY transcription factors. These findings highlight the key role of SmMYB39 in the response of eggplant to salt stress (Jiang et al., 2023).

*R. solanacearum*-resistant, BW2, and susceptible, S86 varieties of eggplant presented upregulated genes regulating the biosynthesis of phenylpropanoids, flavonoids, zeatin, sesquiterpenoids, triterpenoids, and carotenoids. Genes regulating the biosynthesis of salicylic acid (*EGP24622*) and ethylene (*EGP13482* and *EGP06386*) were upregulated in BW2 but downregulated in S86. Jasmonic acid signaling genes (*EGP33594*) and genes regulating ethylene (*EGP30381*) were upregulated in S86, whereas genes (*EGP24622*, *EGP06386* and *EGP13922*) involved in the salicylic acid pathway were downregulated (Yu et al., 2024).

Transcriptomic and metabolomic analyses were conducted on two wild eggplant varieties (LC-2 and LC-7) infected with *V. dahliae*. In LC-2, a highly resistant variety, 6,878 genes were differentially expressed. Genes regulating spermidine and cinnamic acid biosynthesis, cutin biosynthesis, cell wall biogenesis, and other developmental processes were upregulated. In the susceptible variety LC-7, genes governing the biosynthesis of abscisic acid and salicylic acid were differentially expressed. Differential accumulation of defense metabolites such as quinones, flavonoids, terpenoids, lipids, alkaloids, phenolic acids, and amino acids were observed. The accumulation of these metabolites occurred early in the resistant variety (LC-2), whereas the accumulation of these metabolites was delayed in the susceptible variety (LC-7) (Li et al., 2024b) (**Table 4**).

**Table 4:** Differentially expressed genes and affected pathways in eggplant under stress.

Stress type	Cultivar/variety/accession/genotype	Transcription factors/Genes upregulated	Pathways and metabolites upregulated	References
Heat	Tewangda - Tolerant	*CHI3, CYP75A2, PAL2*, *CHSB* and *ASAT*	Anthocyanin biosynthesis	Zhang et al., 2019
	Tewangda - Tolerant	*TPS, P5CS1, GR*, *GPX*, *POD*, *APX, GST*	Trehalose, proline, GR, GPX, POD, APX and GST	Zhang et al., 2020
Salinity Salinity	ML41	SmERFI	ABA-mediated signaling pathways	Shen et al., 2022
		SmMYB39	Nicotinate, nicotinamide, alanine, aspartate, glutamate metabolism	Jiang et al., 2023
Drought	Eggplant	AP2/ERF, bZIP, NAC	Hormone signaling	Villanueva et al., 2023
	*S. dasyphyllum*	AP2/ERF, bZIP, GRAS, bHLH	Ethylene, Abscisic acid, jasmonic acid	
Cold	Fuguhuajiao	*POD*, *GSS*, *GPX*, *GSR*, GST SQS, *CAS*, LAS PP2C, SnRK2	Antioxidant enzymes (POD, GSS, GPX, GSR, GST) Terpenoid synthesis Abscisic acid signaling	Cai et al., 2024
	Fengxiandahongpao	*HK*, *ISA*, *TreY*, *TreZ*, AMY CLH, *SGR1*, *SGR2*	Starch/sucrose metabolism Chlorophyll degradation	
*R. solanacearum*	BW2 – Resistant	*EGP24622, EGP13482* and *EGP06386*	Salicylic acid and ethylene	Yu et al., 2024
	S86 - Susceptible	*EGP33594* and *EGP30381*	Jasmonic acid and ethylene	
*V. dahliae*	LC-2 - Resistant	AP2/ERF, bHLH, GRAS	Hormone signaling, jasmonic acid biosynthesis	Li et al., 2024b
	LC-7 - Susceptible	GARP-G2-like	Hormone signal transduction	

## 8 Conclusion

The current review focused on providing an integrative analysis of eggplant chemodiversity and its functional role in mediating responses to abiotic and biotic stresses. Eggplant is an economically and nutritionally significant vegetable crop. However, this crop is highly susceptible to various abiotic and biotic stresses that significantly impact yield and quality. Secondary metabolites are linked to a plant’s survival strategy, as they are produced in response to specific abiotic and biotic stressors. In eggplant, the accumulation of secondary metabolites enables plants to communicate and respond to stress/external stimuli. These compounds serve as deterrents to pathogens and herbivores and modulate the oxidative stress response under stress conditions. The screening of chemodiversity profiles and their variation in response to different stresses is a useful method for predicting and identifying the number of bioactive compounds linked to phenotypes that have adapted to adverse environmental conditions. Linking phytochemical profiling, stress physiology, and molecular studies has improved our understanding of metabolic pathway regulation and the genetic factors involved in this regulation. This approach can be further used in crop improvement strategies to develop plants with stress tolerant traits.

## 9 Research gaps and future perspectives

Significant advances have been made in the field of chemodiversity. While the metabolite profiles of eggplant fruits have been extensively studied, there is limited information available on the metabolites present in the leaves, stems, and roots. Since the metabolites in these organs also play key roles in whole-plant defense and signaling, identifying them and understanding their role in stress resistance mechanisms is crucial. Furthermore, the precise molecular mechanism through which specific metabolites confer resistance to different stresses are still not fully understood. Future studies can explore metabolite variation induced by domestication and how metabolite-gene associations can be modelled as networks, especially under multiple stress conditions. In addition, high-throughput metabolite screening of eggplants infected with prominent pathogens will enable the identification of genes regulating the synthesis of defense-related secondary metabolites. The identification of key genes involved in metabolite biosynthesis can facilitate marker-assisted selection or genome editing to develop stress-resistant eggplant varieties. The ability of stress-resilient eggplant to produce metabolites with natural pesticidal or fungicidal activity can serve as an eco-friendly alternative to synthetic agrochemicals.
